# Early Mortality of Brain Infarction Patients and Red Blood Cell Distribution Width

**DOI:** 10.3390/brainsci10040196

**Published:** 2020-03-26

**Authors:** Leonardo Lorente, María M. Martín, Pedro Abreu-González, Antonia Pérez-Cejas, Agustín F. González-Rivero, Luis Ramos-Gómez, Mónica Argueso, Jordi Solé-Violán, Juan J. Cáceres, Alejandro Jiménez, Victor García-Marín

**Affiliations:** 1 Intensive Care Unit, Hospital Universitario de Canarias, Ofra s/n, La Laguna, 38320 Santa Cruz de Tenerife, Spain; 2 Intensive Care Unit, Hospital Universitario Nuestra Señora de Candelaria, Crta del Rosario s/n, 38010 Santa Cruz de Tenerife, Spain; mar.martinvelasco@gmail.com; 3 Department of Physiology, Faculty of Medicine, University of the La Laguna, Ofra s/n, La Laguna, 38320 Santa Cruz de Tenerife, Spain; pabreu@ull.es; 4 Laboratory Department, Hospital Universitario de Canarias, Ofra, s/n, La Laguna, 38320 Santa Cruz de Tenerife, Spain; aperezcejas@gmail.com (A.P.-C.); agonriv@hotmail.com (A.F.G.-R.); 5 Intensive Care Unit, Hospital General La Palma, Buenavista de Arriba s/n, Breña Alta, La Palma, 38713 Santa Cruz de Tenerife, Spain; lramosgomez@gmail.com; 6 Intensive Care Unit, Hospital Clínico Universitario de Valencia, Avda, Blasco Ibáñez n°17-19, 46004 Valencia, Spain; moni_begasa@hotmail.com; 7 Intensive Care Unit, Hospital Universitario Dr. Negrín, CIBERES, Barranco de la Ballena s/n, 35010 Las Palmas de Gran Canaria, Spain; jsolvio@gobiernodecanarias.org; 8 Intensive Care Unit, Hospital Insular, Plaza Dr. Pasteur s/n, 35016 Las Palmas de Gran Canaria, Spain; juanjose.caceresagra@gobiernodecanarias.org; 9 Research Unit, Hospital Universitario de Canarias, Ofra s/n, La Laguna, 38320 Santa Cruz de Tenerife, Spain; ajimenezsosa@gmail.com; 10 Department of Neurosurgery, Hospital Universitario de Canarias, Ofra, s/n, La Laguna, 38320 Santa Cruz de Tenerife, Spain; vicgarmar666@gmail.com

**Keywords:** red blood cell distribution width, brain infarction, patients, mortality, prognosis

## Abstract

Background: Meta-analysis has found that high baseline red blood cell distribution width (RDW) is associated with increased long-term mortality (mortality at one year or more) in ischemic stroke. The objectives of this study were to determine whether there is an association between RDW and 30-day mortality, and to explore whether RDW during the first week of ischemic stroke could be a 30-day mortality biomarker. Methods: We included patients with malignant middle cerebral artery infarction (MMCAI). RDW at days 1, 4, and 8 of MMCAI were determined. The end-point study was 30-day mortality. Results: We found that survivor (*n* = 37) in respect to non-survivor patients (*n* = 37) had lower RDW at days 1 (*p* < 0.001), 4 (*p* < 0.001), and 8 (*p* = 0.02). The area under curve (95% CI) for prediction of 30-day mortality by RDW at days 1, 4, and 8 of MMCAI were 0.80 (0.69–0.89; *p* < 0.001), 0.79 (0.66–0.89; *p* < 0.001), and 0.73 (0.58–0.84; *p* = 0.02). Regression analysis showed an association between RDW (odds ratio = 1.695; 95% CI = 1.230–2.335; *p* < 0.001) and 30-day mortality. Conclusions: The association between RDW and early mortality, and the potential role of RDW during the first week of MMCAI as a prognostic biomarker of early mortality were the main novelties of our study.

## 1. Introduction

Red blood cell distribution width (RDW) represents the variability of form and size in red blood cells of a subject and is a hemogram index that is used for the differential diagnosis of anemia [1]. High RDW has been associated with increased mortality in patients with coronary disease [2], cardiac arrest [3], heart failure [4], pancreatitis [5], liver disease [6], sepsis [7,8], or traumatic brain injury [9].

RDW has been previously scarcely explored in patients with ischemic stroke [10,11,12,13,14]. In a recently published meta-analysis, it has been found that high baseline RDW is associated with increased mortality in ischemic stroke, over all long-term mortality (mortality at one year or more) [10]. In some studies included in the meta-analysis, the end-point was long-term mortality (mortality at one year or more); in two studies it was in-hospital mortality (but the day of death was not reported) [11,12], in one study it was 90-day mortality [13], and in one study it was 30-day mortality [14]. However, in the study by Duchnowsi et al. the aim was to evaluate the prognostic value of RDW for ischemic stroke in the early postoperative period in patients undergoing valve replacement. Only 14 patients developed perioperative ischemic stroke, and only 4 patients were dead at 30 days; therefore, is difficult to establish the role of RDW for 30-day mortality prediction in ischemic stroke [14]. Thus, there is not conclusive data on the role of RDW for early mortality prediction in ischemic stroke, and the objectives of this study were to compare RDW during the first week of brain infarction in 30-day surviving and non-surviving patients, to determine whether there is an association between RDW and 30-day mortality, and to explore whether RDW during the first week of brain infarction could be a 30-day mortality biomarker.

## 2. Methods

### 2.1. Design and Subjects

This prospective and observational study was performed in six Spanish hospitals with the approval of the Institutional Board of each hospital (CHUC-2009-46): H. General de La Palma, H. Universitario Dr. Negrín (Gran Canaria), H. Universitario Nuestra Señora de Candelaria (Tenerife), H. Insular (Gran Canaria), H. Clínico Universitario de Valencia, and H. Universitario de Canarias (Tenerife). Additionally, the inclusion of patients was performed with the written and signed consent of a relative of each patient.

Patients with malignant middle cerebral artery infarction (MMCAI) were included. We established the diagnosis of MMCAI with the presence of an acute middle cerebral artery infarction with parenchymal hypodensity in at least of 50% of this territory and midline shift in the computed tomography, and with the presence of acute neurological deterioration consisting at least of Glasgow Coma Scale (GCS) < 9 [15]. We excluded patients with malignant disease, comfort measures only, age less than 18 years, or inflammatory disease.

We collected sex, age, and the history of chronic obstructive pulmonary disease (COPD), diabetes mellitus, heart failure, chronic renal failure, and arterial hypertension. In addition, we registered body temperature, GCS, Acute Physiology and Chronic Health Evaluation II (APACHE II) score [16], bilirubin, sodium, lactic acid, creatinine, glycemia, fraction of inspired oxygen (FIO_2_), partial pressure of arterial oxygen (PaO_2_), platelets, leukocytes, fibrinogen, hemoglobin, international normalized ratio (INR), activated partial thromboplastin time (aPTT), hemorrhagic transformation, volume infarction, midline shift, and the realization of decompressive craniectomy.

Our end-point study was mortality at 30 days. In addition, we collected RDW and blood levels of tumor necrosis factor (TNF)-alpha and malondialdehyde at days 1, 4, and 8 of MMCAI.

### 2.2. Statistical Methods

We used frequencies (percentages) and medians (25th–75th percentiles) to report categorical and continuous variables. The Chi-square test and Wilcoxon–Mann–Whitney test were used to compare categorical and continuous variables between survivor and non-survivor patients at 30 days. The capacity by RDW at days 1, 4, and 8 of MMCAI for 30-day mortality prediction was determined by receiver operating characteristic (ROC) analyses; and we reported specificities and sensitivities for the cut-offs of RDW (using Youden J index) at days 1, 4, and 8. To test the association between RDW and 30-day mortality, controlling for GCS, lactic acid, and platelet count was carried out using a multiple logistic regression analysis with the enter method. To determine the existence of an association between continuous variables, we used the coefficient of Spearman’s rank correlation. The programs LogXact 4.1 (Cytel Co., Cambridge, MA, USA), SPSS 17.0 (SPSS Inc., Chicago, IL, USA), and NCSS 2000 (Kaysville, UT, USA) were used in the statistical analyses. A difference with *p*-value < 0.05 was considered statistically significant.

## 3. Results

We found that the 37 survivor patients in respect to the 37 non-survivor patients had higher GCS and lower lactic acid (Table 1). We found higher RDW at days 1 (*p* < 0.001), 4 (*p* < 0.001), and 8 (*p* = 0.02) in non-survivor than in survivor patients (Table 2). In addition, we found higher blood levels (*p* < 0.001) of TNF-alpha and malondialdehyde at days 1, 4, and 8 in non-survivor than in survivor patients (Table 2).

**Figure 1:**
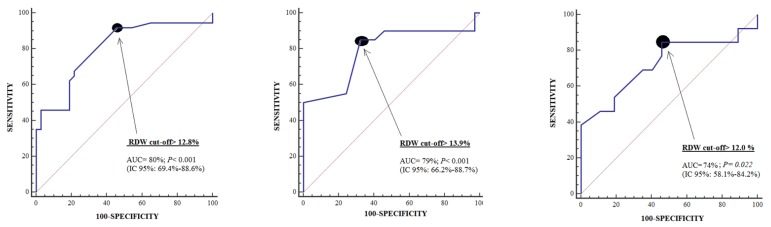
Receiver operation characteristic (ROC) curve using red blood cell distribution width (RDW) at days 1, 4, and 8 as predictor of mortality at 30 days.

The area under curve (95% confidence interval) for prediction of 30-day mortality by RDW at days 1, 4, and 8 of MMCAI were 0.80 (0.69–0.89; *p* < 0.001), 0.79 (0.66–0.89; *p* < 0.001), and 0.73 (0.58–0.84; *p* = 0.02) respectively (Figure 1). Table 3 showed specificities and sensitivities for the cut-offs of RDW at days 1, 4, and 8 of MMCAI.

Regression analyses showed an association between RDW (odds ratio = 1.695; 95% CI = 1.230–2.335; *p* = 0.001) and 30-day mortality, controlling for lactic acid, GCS, and platelet count (Table 4). We found a positive association between RDW and serum TNF-alpha levels at days 1 (rho = 0.54; *p* < 0.001), 4 (rho = 0.45; *p* < 0.001), and 8 (rho = 0.50; *p* < 0.001) of MMCAI. Additionally, we found a positive association between RDW and serum malondialdehyde concentrations at days 1 (rho = 0.46; *p* < 0.001), 4 (rho = 0.40; *p* = 0.002), and 8 (rho = 0.48; *p* < 0.001) of MMCAI.

## 4. Discussion

Previously, in a recently published meta-analysis, it has been found that high baseline RDW is associated with increased mortality in ischemic stroke, over all long-term mortality (mortality at one year or more) [10]. However, there is not conclusive data on the role of RDW for early mortality prediction in ischemic stroke. A study by Duchnowsi et al. observed 14 patients developing perioperative ischemic stroke, of which four patients died during the first 30 days, which showed it is difficult to establish 30-day mortality prediction by RDW in ischemic stroke [14]. Thus, that non-survivors showed higher RDW during the first week of MMCAI showed that RDW at any time during the first week of MMCAI could be used as biomarker of early mortality, and that there is an association between RDW and early mortality according to the regression analysis, are three interesting novelties of our study. We think that all new findings could help physicians in the estimation of early prognosis in those patients.

We found that non-survivor patients had higher RDW and lactic acid, and lower GCS and platelet count. However, only the RDW and GCS of them were associated with mortality in the regression analysis. We included only those four variables in the multiple logistic regression analysis, due to the fact that we registered 37 deaths in our series, and those variables showed the highest differences between non-survivor and survivor patients. We have not included serum levels of TNF-alpha and malondialdehyde in the regression analysis due to the fact that we found an association between RDW, TNF-alpha, and malondialdehyde.

Different factors have been associated with the outcome stroke such as oxidation [17,18], inflammation [19,20,21,22], hyperglycemia [23], or alterations in brain perfusion due to blood pressure variability [24,25]. In addition, there has been found the association of RDW with oxidative stress in animal models [26], with blood TNF-alpha levels in critically ill patients [27,28], and with atrial fibrillation and its risk of complications and mortality [29,30,31]. Another novel finding of our study was that there is a positive association between RDW and blood levels of malondialdehyde and TNF-alpha during the first week of MMCAI. In summary, we found that non-survivor patients showed higher RDW, higher blood malondialdehyde levels, and higher blood TNF-alpha levels during the first week of TBI, and that those parameters are associated. We think that the association between mortality and RDW in MMCAI patients could be due to a higher oxidative state and a higher inflammation state.

Some limitations of our study was that data on folate, iron, vitamin B12, blood smear, and reticulocyte count to complete the description of red blood cell has been not reported. In addition, we have not registered some factors that could be associated with outcome stroke and RDW as atrial fibrillation and blood pressure variability. However, we think that our study has some strengths, as we reported data on RDW, malondialdehyde levels, and TNF-alpha levels during the first week of MMCAI.

We believe that there are multiple factors associated with outcome stroke, many of them are easily obtainable (blood biomarkers of inflammatory and oxidation) and may be incorporated into clinical scoring systems to improve outcome prediction. In addition, many of those factors are modifiable and can become targets of therapeutic interventions. In this respect, we believe that the use of RDW could be standardized for early mortality prediction in MMCAI due to the fact that RDW is a laboratory parameter that is automatically in conventional hemograms.

## 5. Conclusions

The association between RDW and early mortality, and the potential role of RDW during the first week of MMCAI as a prognostic biomarker of early mortality were the main novelties of our study.

## Figures and Tables

**Table 1:** Characteristics of 30-day surviving and non-surviving patients.

	Survivors (*n* = 37)	Non-Survivors (*n* = 37)	*p*-Value
Age (years): median (p 25–75)	60 (47–68)	61 (53–70)	0.50
Female: *n* (%)	14 (37.8)	14 (37.8)	0.99
Heart failure: *n* (%)	1 (2.7)	1 (2.7)	0.99
Diabetes mellitus: *n* (%)	5 (13.5)	9 (24.3)	0.37
COPD: *n* (%)	1 (2.7)	1 (2.7)	0.99
Chronic renal failure: *n* (%)	2 (5.4)	2 (5.4)	0.99
Arterial hypertension: *n* (%)	21 (56.8)	19 (51.4)	0.82
GCS score: median (p 25–75)	8 (6–8)	6 (3–7)	0.01
APACHE-II score: median (p 25–75)	20 (16–25)	22 (19–27)	0.07
Lactic acid (mmol/L): median (p 25–75)	1.20 (0.90–1.70)	1.60 (1.01–2.88)	0.03
Temperature (°C): median (p 25–75)	36.4 (36.0–37.0)	36.9 (36.0–37.2)	0.10
Bilirubin (mg/dL): median (p 25–75)	0.60 (0.42–0.80)	0.65 (0.35–1.13)	0.85
Glycemia (g/dL): median (p 25–75)	127 (102–170)	136 (113–161)	0.50
Creatinine (mg/dL): median (p 25–75)	0.80 (0.65–1.10)	1.00 (0.70–1.20)	0.21
Sodium (mEq/L): median (p 25–75)	139 (136–143)	140 (138–143)	0.50
PaO_2_ (mmHg): median (p 25–75)	144 (104–285)	115 (94–267)	0.40
PaO_2_/FIO_2_ ratio: median (p 25–75)	293 (204–366)	248 (188–320)	0.18
INR: median (p 25–75)	1.06 (1.00–1.20)	1.15 (1.01–1.31)	0.05
aPTT (seconds): median (p 25–75)	28 (25–30)	27 (26–32)	0.99
Platelets: median × 10^3^/mm^3^ (p 25–75)	200 (170–267)	173 (134–212)	0.02
Fibrinogen (mg/dL): median (p 25–75)	445 (415–526)	419 (339–612)	0.90
Leukocytes: median × 10^3^/mm^3^ (p 25–75)	12.2 (9.5–17.0)	13.8 (9.3–17.7)	0.40
Hemoglobin (g/dL): median (p 25–75)	12.2 (11.4–14.5)	12.5 (11.0–14.8)	0.97
Thrombolysis: *n* (%)	12 (32.4)	12 (32.4)	0.99
Hemorrhagic transformation: *n* (%)	8 (21.6)	8 (21.6)	0.99
Volume infarction (mL): median (p25–75)	181 (105–235)	190 (65–288)	0.72
Midline shift (mm): median (p 25–75)	6.5 (2.8–11.2)	10.0 (4.0–15.0)	0.41
Decompressive craniectomy: *n* (%)	9 (24.3)	7 (18.9)	0.78

p 25–75 = percentile 25th–75th; COPD = Chronic Obstructive Pulmonary Disease; GCS = Glasgow Coma Scale; APACHE II = Acute Physiology and Chronic Health Evaluation II; PaO_2_ = pressure of arterial oxygen/fraction inspired oxygen; FIO_2_ = pressure of arterial oxygen/fraction inspired oxygen; INR = international normalized ratio; aPTT = activated partial thromboplastin time.

**Table 2:** Red blood cell distribution width (RDW), malondialdehyde, tumor necrosis factor (TNF)-alpha at days 1, 4, and 8.

Parameters	Survivors	Non-Survivors	*p*-Value
Day 1	(*n* = 37)	(*n* = 37)	
RDW: median % (percentile 25–75)	12.7 (11.2–13.2)	13.9 (13.0–17.0)	<0.001
Malondialdehyde: median nmol/mL (percentile 25–75)	1.76 (1.39–2.24)	2.99 (2.08–4.17)	<0.001
TNF-alpha median pg/mL (percentile 25–75)	9.8 (9.2–11.3)	15.5 (13.2–16.7)	<0.001
Day 4	(*n* = 37)	(*n* = 20)	
RDW: median % (percentile 25–75)	12.0 (10.3–14.5)	15.1 (14.0–17.1)	<0.001
Malondialdehyde: median nmol/mL (percentile 25–75)	1.64 (1.37–1.90)	2.95 (2.50–3.19)	<0.001
TNF-alpha median pg/mL (percentile 25–75)	9.8 (9.1–10.9)	14.9 (13.3–16.2)	<0.001
Day 8	(*n* = 37)	(*n* = 13)	
RDW: median % (percentile 25–75)	11.5 (9.9–14.0)	14.9 (12.7–16.9)	0.02
Malondialdehyde: median nmol/mL (percentile 25–75)	1.46 (1.19–1.92)	2.71 (2.52–2.88)	<0.001
TNF-alpha: median pg/mL (percentile 25–75)	9.3 (8.9–10.4)	14.8 (13.5–17.2)	<0.001

RDW: Red blood cell distribution width; TNF: tumor necrosis factor.

**Table 3:** Thirty-day mortality prognostic capability of red blood cell distribution width (RDW) at days 1, 4, and 8 of malignant middle cerebral artery infarction.

	Day 1	Day 4	Day 8
Cut-off of RDW in %	>12.8	>13.9	>12.0
Specificity (95% confidence interval)	54% (37%–71%)	68% (50%–82%)	54% (37%–71%)
Sensitivity (95% confidence interval)	92% (78%–98%)	85% (62%–97%)	85% (55%–98%)

**Table 4:** Multiple logistic regression analysis to predict 30-day mortality.

Variable	Odds Ratio	95% Confidence Interval	*p*
Platelet count (each 1000/mm^3^)	0.995	0.987–1.003	0.22
Lactic acid (mmol/L)	1.148	0.642–2.053	0.64
Glasgow Coma Scale (points)	0.661	0.480–0.910	0.01
RDW (%)	1.695	1.230–2.335	0.001

RDW: red blood cell distribution width.
